# A Few Abnormal Results Following Dehorning Cattle

**Published:** 1894-12

**Authors:** J. D. Nichbert

**Affiliations:** Assistant State Veterinarian of Illinois


### A FEW ABNORMAL RESULTS FOLLOWING DEHORNING CATTLE.

By Dr. J. D. Nichbert,
Assistant State Veterinarian of Illinois.

CASE I.

Spotted steer, one year old, dehorned three months previous with saw. Emaciated, appetite fairly good, large swelling above right orbit extending across forehead, small sinus where horn was removed, which discharged a fetid pus. Diagnosis, abscess of frontal sinus. Cast and secured steer, operated by trephining above right orbit, found cavity filled with a mass of cheesy-like pus, which was removed by copious injection of warm water and lysol, 2 per cent. solution; ordered daily injections of same, which was continued for six days, when discharge almost entirely ceased, patient rapidly improved; injections discontinued after ten days, when opening closed, frontal bones gradually assumed normal shape, and steer made good recovery.

CASE II.

Red steer, three years old, weight about thirteen hundred pounds, being fed for market. First noticed by owner standing away from rest of herd, motionless head, turned to one side, apparently suffering intense pain; after lapse of twenty-four hours, steer in same condition, taking no nourishment, would move only when being driven about feed yard. Owner becoming alarmed, sent for me; upon making inquiry, ascertained that steer had been dehorned six months previously, and had apparently done as well as rest of herd after the operation, until forty-eight hours previous, when above symptoms were noticed. Caught and secured patient; upon examination found slight elevation of temperature, breathing accelerated, slight enlargement above left orbit, small opening where horn was removed. Diagnosis, same as Case I. As the owner would not consent to trephining, I enlarged opening where horn was sawed off, when about four ounces of serum and pus escaped, along with some particles of sawdust and hairs that had dropped into cavity when horn was removed. Thoroughly cleansed cavity with warm water and lysol, 2 per cent. solution, and released patient. Results: relief almost instantaneous, steer began eating in one hour; was sent to market in one week, although there was yet some discharge from opening.

CASE III.

Aged cow; roan; symptoms similar to Case II, only more marked; cow had stood with head turned to right, back arched, coat staring; had refused all feed for four days; very weak; hard, bony enlargement above left eye. Trephined sinus two inches above left eye. Copious discharge of pus and hairs, with some pieces of straw and dirt. Thoroughly cleansed cavity with 2 per cent. lysol solution. Cow began eating as soon as released; ordered daily injections into cavity, of warm water and lysol, as above. Results: good, rapid recovery.

As these are but few of the many such cases as have come under my observation since dehorning has become almost universal in this section, I came to the following conclusions: That in many instances there is too much of the tissues removed, or, in other words, there is too much of the bone removed with the horn, thus leaving a large opening, which, being left open by the operator, catches dirt and foreign substances before it closes, and as there is some itching during healing, the animal is inclined more or less to rub the head against straw or hay stacks, and thus get pieces of the same into the cavities. Where the sinus is large it should be plugged with oakum and tar, or some other medicant, and not be left open to admit cold air, dust and dirt, which create irritation, and ultimately lead to abscesses and death, if not relieved.

